# Does D-Cycloserine Enhance Exposure Therapy for Anxiety Disorders in Humans? A Meta-Analysis

**DOI:** 10.1371/journal.pone.0093519

**Published:** 2014-07-03

**Authors:** Helga Rodrigues, Ivan Figueira, Alessandra Lopes, Raquel Gonçalves, Mauro Vitor Mendlowicz, Evandro Silva Freire Coutinho, Paula Ventura

**Affiliations:** 1 Institute of Psychiatry, Universidade Federal do Rio de Janeiro (IPUB-UFRJ), Rio de Janeiro, Brazil; 2 Department of Psychiatry and Mental Health, Universidade Federal Fluminense (MSM-UFF), Niteroi, Brazil; 3 Department of Epidemiology, Escola Nacional de Saúde Pública (ENSP-FIOCRUZ) Rio de Janeiro, Brazil; 4 Institute of Psychology, Universidade Federal do Rio de Janeiro (IPUB-UFRJ), Rio de Janeiro, Brazil; University of Wuerzburg, Germany

## Abstract

The treatment of anxiety is on the edge of a new era of combinations of pharmacologic and psychosocial interventions. A new wave of translational research has focused on the use of pharmacological agents as psychotherapy adjuvants using neurobiological insights into the mechanism of the action of certain psychological treatments such as exposure therapy. Recently, d-cycloserine (DCS) an antibiotic used to treat tuberculosis has been applied to enhance exposure-based treatment for anxiety and has proved to be a promising, but as yet unproven intervention. The present study aimed to evaluate the efficacy of DCS in the enhancement of exposure therapy in anxiety disorders. A systematic review/meta-analysis was conducted. Electronic searches were conducted in the databases ISI-Web of Science, Pubmed and PsycINFO. We included only randomized, double-blind, placebo-controlled trials with humans, focusing on the role of DCS in enhancing the action of exposure therapy for anxiety disorders. We identified 328 references, 13 studies were included in our final sample: 4 on obsessive-compulsive disorder, 2 on panic disorder, 2 on social anxiety disorder, 2 on posttraumatic stress disorder, one on acrophobia, and 2 on snake phobia. The results of the present meta-analysis show that DCS enhances exposure therapy in the treatment of anxiety disorders (Cohen d =  −0.34; CI: −0.54 to −0.14), facilitating the specific process of extinction of fear. DCS seems to be effective when administered at a time close to the exposure therapy, at low doses and a limited number of times. DCS emerges as a potential new therapeutic approach for patients with refractory anxiety disorders that are unresponsive to the conventional treatments available. When administered correctly, DCS is a promising strategy for augmentation of CBT and could reduce health care costs, drop-out rates and bring faster relief to patients.

## Introduction

Anxiety disorders are the most prevalent mental disorders, in the United States for example, more than 30 million Americans have experienced at least one anxiety disorder in their lifetime [1]. They often present a chronic course and are as disabling as physical diseases [2], significantly compromising the quality of life [3]. They also present high rates of comorbidity with other mental and chronic physical problems [4]. Anxiety disorders have a significant economic impact, leading to marked reduction in productivity and generating high medical and social costs. In the U.S., the direct and indirect cost of anxiety disorders was estimated at 42 billion dollars per year [5]. It is predicted that in 2020 anxiety disorders, together with depressive disorders, will be the second most common disease in the world [6].

Cognitive-behavioral therapy (CBT) and pharmacotherapy represent the front-line interventions for anxiety disorders and exposure is considered the gold standard behavioral intervention. Selective serotonin reuptake inhibitors (SSRIs) are the treatment of first choice for anxiety disorders according to most guidelines and algorithms involving pharmaceutical drugs [7]. The combined treatment of CBT with pharmacotherapy does not appear to present additional benefits as compared to CBT alone for some anxiety disorders [8], such as panic disorder, obsessive-compulsive disorder (OCD) and social phobia [9]; [10]; [11]. Even after first choice treatments, the disorder does not remit completely in a high proportion of patient who still require additional treatment [12]; [13]. Over 40% of patients responding partially to antidepressants suffer from clinically significant residual symptoms [14]. Moreover, a considerable number of patients discontinue treatment for because of the adverse effects of conventional pharmacotherapy. For example, SSRIs induce many undesired effects in the sphere of sexuality, sleep and weight, resulting in treatment interruption or failure to use the correct therapeutic doses [15]. With regard to psychotherapy, 50% of the patients do not respond or abandon the conventional treatment with CBT [16]. Furthermore, many patients do not adhere to the CBT treatment due to the anxiety generated by the exposure technique, which is an essential part of the treatment for anxiety disorders [17]. In this context, it is necessary to develop new effective strategies for the treatment of patients resistant and/or intolerant to the current treatments.

In translational research, the antibiotic D-cycloserine (DCS), a glutamatergic agent, partial agonist at the glycine recognition site of the *N-*methyl-D-aspartate (NMDA) receptor in the amygdala, emerged as part of a new strategy using a combination of pharmacotherapy and CBT. Originally designed to be a tuberculostatic agent, DCS was later used as a potential treatment for Alzheimer’s dementia and negative symptoms in schizophrenia [18]; [19]. The traditional pharmacological treatment for mental disorders requires the correction of hypothetical biochemical abnormalities in critical structures of the central nervous system. Anxiety disorders have a component of emotional learning. DCS seems to exert an effect on the emotional learning component, accelerating the process of associative learning and contributing to an improvement in symptoms [20]. Studies with animal models strongly suggest that DCS facilitates the process of extinction of conditioned fear [21]. On the other hand, antagonists at the glutamatergic NMDA receptor, which is linked to learning and memory, seem to block learning of extinction of fear. DCS would have a role in enhancing the learning of extinction of fear, a central mechanism in exposure therapy.

The aim of this paper was to perform a systematic review and a meta-analysis of the efficacy of DCS as a strategy of augmentation of CBT in patients with anxiety disorders.

## Methods

### Search strategy

Electronic searches were performed in the following databases: ISI-Web of Science, Pubmed/Medline and PsycINFO, covering the period from 1974 to November 23, 2012. Articles published in any language were considered. The following terms were used:

### ISI (advanced search)

• TS  =  (“behavio*therapy” OR “cognitive behavio*therapy” OR “exposure therapy” OR “exposure treatment” OR “exposure-based behavior therapy” OR CBT OR flooding OR virtual reality).
• TS  =  (d-cycloserine OR DCS).

All of the 3 citation databases were activated (Science Citation Index Expanded (SCI-EXPANDED), Social Sciences Citation Index (SSCI) and Arts & Humanities Citation Index (A&HCI), and we restricted the search criteria in order to include only “articles” and notes.

### Pubmed/Medline (advanced search)

• Cycloserine OR d-cycloserine OR DCS.
AND.
• “behavior therapy” OR “behaviour therapy” OR “exposure therapy” OR “exposure treatment” OR “exposure-based” OR “implosive therapy” OR flooding OR “cognitive therapy” OR “cognitive behavior therapy” OR “cognitive behaviour therapy” OR “cognitive-behavior therapy” OR CBT.

### PsycINFO

• “behavio*therapy” OR “cognitive behavio*therapy” OR “exposure therapy” OR “exposure treatment” OR “exposure-based behavior therapy” OR CBT OR flooding OR “virtual reality”.
• d-cycloserine OR DCS.

In these databases, the terms were searched directly in advanced search (All Fields) and the results of each individual search were combined. APA Books were excluded.

Besides electronic searches, manual searches were also performed through bibliographical references. We also consulted with experts in the field about the existence of additional studies that could not be located through the electronic search.

### Inclusion/exclusion criteria

**Figure 1:**
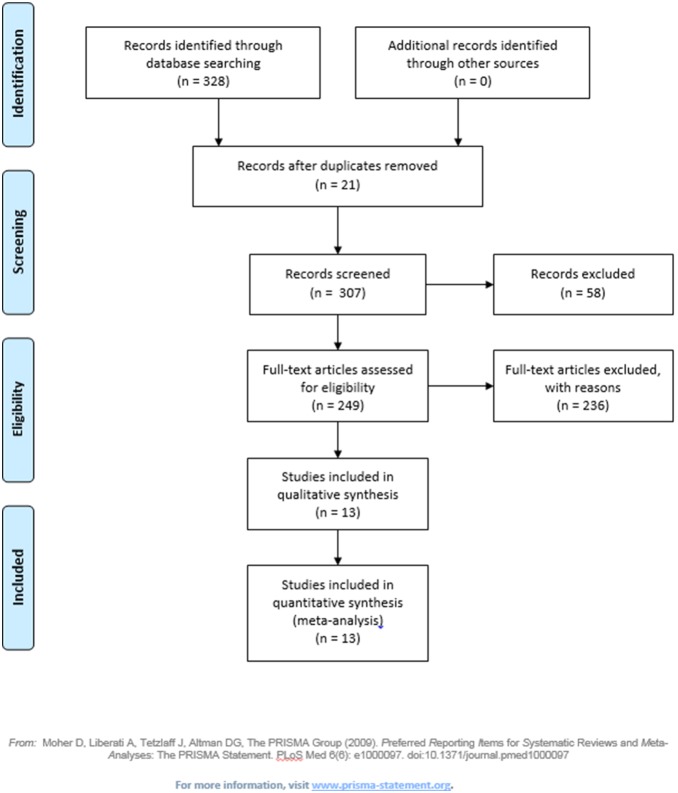
Flow Diagram.

We included “articles” and “notes” and excluded “review articles”, book chapters and dissertations. Our search was based solely on randomized, double-blind, placebo-controlled trials of D-cycloserine augmentation of behavioral therapy for the treatment of anxiety disorders (with or without comorbidity with others mental disorders) in a human population. We included articles focused on enhancement of exposure therapy through DCS used during and/or at times close to the exposure treatment. Studies that did not focus on CBT (for example, that investigated the neural mechanisms of the process of extinction with DCS) and that did not include anxiety disorders according to the criteria of the Diagnostic and Statistical Manual of Mental Disorders (DSM) III, III-R, IV and IV-TR [22]; [23] were excluded. Studies duplicated and listed twice were excluded. Figure 1 shows the study selection process. Table 1 shows the selected studies.

**Table 1:** The selected studies.

	NCBT +DCS/PCB+ CBT	Disorder	Demographic Age(mean) % ofwomen	DCS Dose(mg) Time Before CBT	CBT protocol	Results
Storch et al.2007	12/12	OCD	29.0 ± 9.9 50%	250 4 hours	12 weeklysessions of ET	There was no clinically or statistically significant difference between the groups and no difference in remission: 42% (E/RP + DCS) and 58% (E/RP + PCB). There was no difference between groups on CGI: 83% (E/RP + DCS) and 92% (E/RP + PCB) were considered responders.
Kushner et al.2007	14/11	OCD	Not shown	125 Approximately 2 hours	Up to 10 ET, twice weekly	DCS group showed faster reduction of obsession-related fears (Y-BOCS and SUDS). >50% reduction on SUDS in all items of the hierarchy in two sessions earlier than the placebo group.
Wilhelm et al.2008	10/12	OCD	Not shown	100 1 hour	1 psychoeducational/treatmentplanning session(90 minutes) +10 behavior therapy sessions (60 minutes each) held twice a week	DCS group had significant improvement in OCD symptoms on Y-BOCS compared to placebo group at mid-treatment. There was no statistically significant difference between groups after treatment and at one-month follow-up.
Storch et al. 2010	15/15	OCD	12.2 ± 2.8 63%	25 mg 1 hour(4–10 sessions)	Ten 60-min CBT sessions	Moderate (72% - CBT + DCS) and small (58% - CBT + PB) effect size on CYBOCS. Moderate effect size on CGI-S, in support of CBT + DCS with a 57% versus 41% symptom reduction.
Otto et al., 2010	15/12	Panic Disorder	35.0 ± 11.0 50%	50 1 hour(sessions 3–5)	5 sessions of cognitive-behavior therapy	Better results on PDSS and CGI-S and clinically significant changes (77% DCS vs. 33% PCB). Large effect size.
Siegmund et al., 2011	20/19	AgoraphobiaPanic Disorder	37.85 ± 11.3 (DCS) 37.32 ± 13.0 (PCB) 46%	50 1 hour	11 sessions (90 minutes each) of CBT in group	There was no statistical difference between DCS and placebo groups. DCS accelerated reduction of symptoms with in vivo exposure therapy in more severe patients in total score of Panic and Agoraphobia Scale.
Hofmann et al. 2006	12/15	Social Anxiety Disorder	33.70 ± 10∶02 29.62%	50 1 hour (2 to 5 sessions)	5 sessions of individual or group therapy exposure	Group receiving DCS reported significant decrease in anxiety and better social outcomes measured by SPAI, LSAS, and CGI-S.
Guastella et al. 2008	28/28	Social Anxiety Disorder	29.0 ± 8.1 63%	50 1 hour	5 sessions of group ET	DCS enhanced the exposure therapy. Reductions in GAF, SPAI, LSAS, BFNE and LIS, and improvements maintained at one-month follow-up.
Kleine et al. 2012	24/21	PTSD	36.27 ± 11∶56 (DCS)40.26 ± 11.05 (PCB)Not shown	50 1 hour	10 weekly exposure sessions	DCS seems to have enhanced the effects of treatment, but patients who received DCS showed stronger response to therapy. DCS showed greater reduction of symptoms in participants who had more severe pre-treatment PTSD and needed longer treatment.
Litz et al. 2012	13/13	PTSD	32.77 ± 9.85 (DCS)31.62 ± 9.10(PCB) Not shown	50 30 min(sessions 2–5)	6 sessions of 60–90 minof exposure therapy	DCS failed to show an overall augmentation effect: 36.4% of the completers in placebo group and 33.3% of those in the DCS condition no longer met criteria for PTSD on CAPS. 50% of the completers met the criteria for status responders: 70% of the placebo group and 30% of the DCS group.
Ressler et al. 2004	17/10	Acrophobia	46.4 ± 2.8 (DCS)44.8 ± 2.3 (PCB) 59.26%	50/500“Acutely prior to psychotherapy”	2 sessions of VRE therapy	There was no significant difference between the 50 mg and 500 mg groups. DCS group showed higher percentages of subjects who reported “much improvement” or “very much improvement”. Reductions in number of skin conductance fluctuations and greater improvements in measures of symptoms of acrophobia in the real world and improvements maintained at 3-month follow-up.
Tart et al., 2012	15/14	Acrophobia	29.33 ± 14.67 (DCS)37.71 ± 16.81 (PCB)Not shown	50 After each session	Two-session protocol VRE	“There was clinical improvement in all outcome measures, but there was no significant statistical difference between the groups on DCS versus placebo. 81.8% of placebo group and 66.7% of DCS responded. 63.5% of placebo group and 60.0% of DCS remitted.
Nave et al., 2012	10/10	Snake Phobia	34.60 ± 12.69 (DCS)39.00 ± 13.91 (PCB)60%	50 1 hour	Single session ofgraded exposuretherapy	Reduction in both groups on Snake Questionnaire, although DCS group achieved faster reduction in the hierarchy.

ADIS-IV  =  Anxiety Disorders Interview Schedule for DSM-IV; BFNE  =  Brief Fear of Negative Evaluation Scale; CGI-S  =  Clinical Global Improvement Severity; CYBOCS  =  Children’s Yale-Brown Obsessive-Compulsive Scale; DCS  =  D-cycloserine; ET  =  exposure therapy; GAF  =  Global Assessment of Functioning; LIS  =  Life Interference Scale; LSAS  =  Liebowitz Social Anxiety Scale; PCB  =  placebo; Y-BOCS  =  Yale-Brown Obsessive Compulsive Scale; PDSS  =  Panic Disorder Severity Scale; SPAI  =  Social Phobia and Anxiety Inventory; PTSD  =  Post-traumatic Stress Disorder; SUDS  =  Subjective Unit of Distress Scale.

### Quality evaluation of the studies and statistical analysis

**Figure 2:**
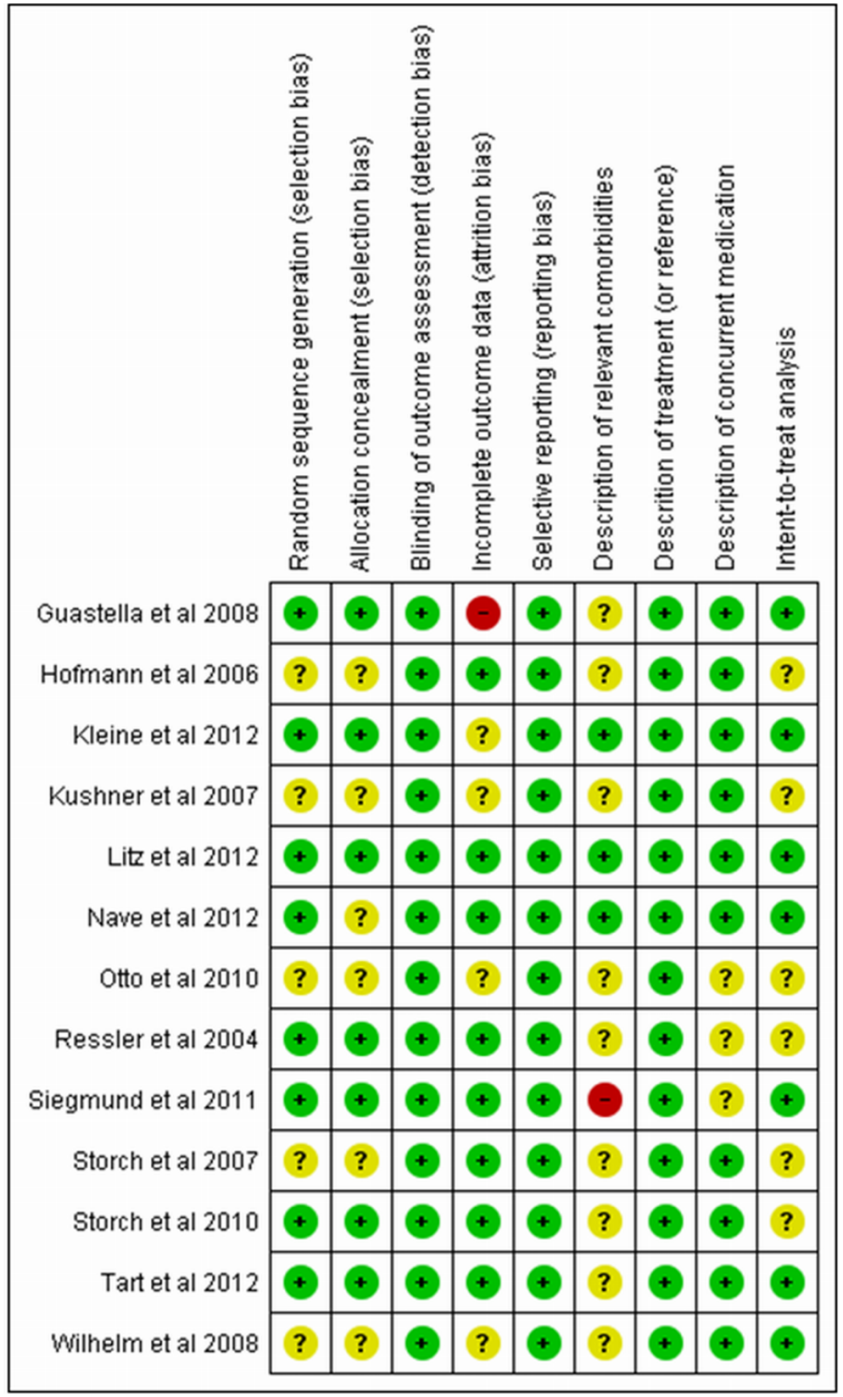
Methodological quality of the included studies (by study).

**Figure 3:**
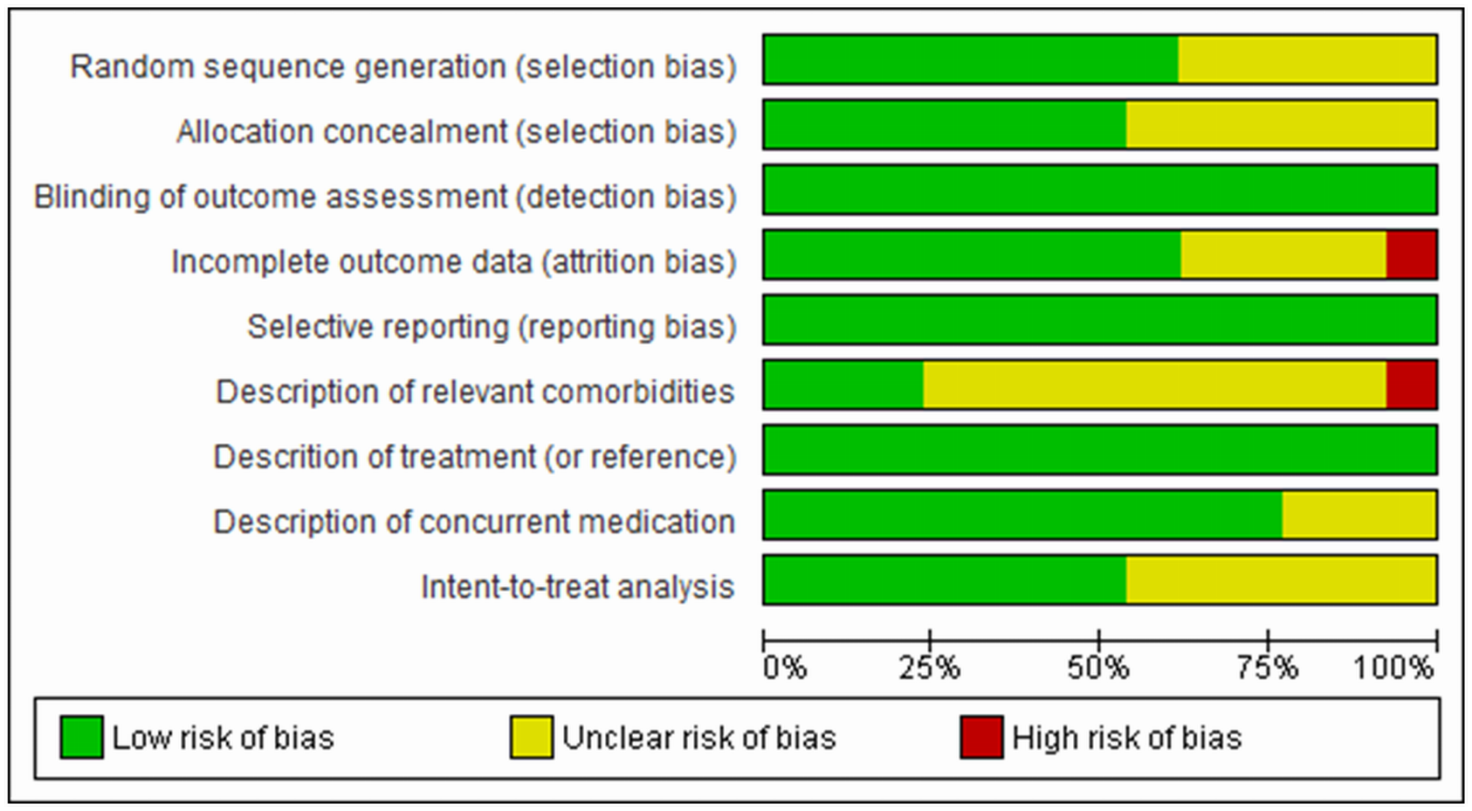
Methodological quality of the included studies (by domain).

The use of scales with summary scores for assessing risk of bias has been criticized and discouraged. For this reason we decide to use an adapted model of the graphs proposed by the Cochrane Collaboration to evaluate the methodological quality of the studies included in this review [24]. For this evaluation we mainly took into consideration the following items: randomization, allocation concealment, blinding, selective reporting and type of analysis. Each of these items was classified (Figure 2 and figure 3) as “low risk of bias”, “high risk of bias” or unclear (when there was not sufficient information).

**Figure 4:**
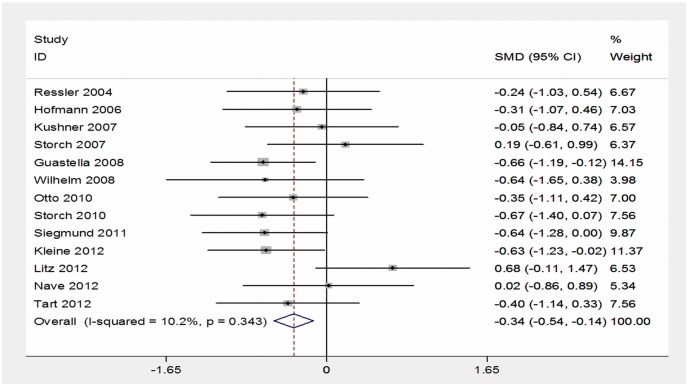
Forest-plot.

Because studies have made use of different scales for anxiety disorders, we estimated the difference of standardized means to obtain the summary measure (effect size) using fixed effects models. Thus, the differences between the final scores in the intervention group and in the control group were expressed in number of standard deviations. Negative values indicate/favor the intervention group. Standardized effect sizes (individual and pooled) were presented using a forest-plot. The heterogeneity between the results of the studies was assessed using the chi-square test for heterogeneity and I^2^ statistic. The I^2^ represents the proportion of the total variance that is due to inter-study variation (heterogeneity). Values below 30% suggest a low variability in the results across studies (homogeneity) [25]. Analyses were performed using Stata 12. See figure 4.

## Results

Our search of the three databases identified 328 publications from which thirteen studies were selected for this review (Figure 1). Most participants had at least one additional DSM Axis I diagnosis and were taking a stable dose of psychotropic medications, which were maintained during the studies. The results of the selected studies were divided by the primary anxiety disorders. Of the thirteen studies identified, four described the treatment of patients with obsessive-compulsive disorder (OCD), two with panic disorder, two with social anxiety disorder, two with post-traumatic stress disorder (PTSD) and three with simple phobia. Table 1 describes the main characteristics of these studies.

Among the four studies mainly focused on patients with OCD, three found no positive effects for enhancement of exposure therapy with DCS (two found positive results only in mid-treatment) and one reported moderate effect. At least half of the participants were taking psychiatric medications on stable doses just before and during the study. All participants with OCD were diagnosed using the Structured Clinical Interview for DSM-IV [23].

In the only study with children, Storch et al. [26] randomized 30 young people to ten 60-min CBT sessions plus 25 mg of DCS or placebo taken before sessions 4 through 10. The included participants had a diagnosis of OCD, established according to score ≥16 on the Children’s Yale-Brown Obsessive Compulsive Scale (CYBOCS) [27], and were stable on any psychotropic medications for 12 weeks. Evaluations were performed at the end of treatment, after session 6, and within one week post-treatment. Children randomized to DCS augmentation of CBT showed moderate effect size relative to a placebo control on several symptoms severity indexes. For the Clinical Global Impressions-Severity Scale (CGI-S) [28], the effect size was moderate in support of CBT + DCS with a 57% versus 41% symptom reduction. For the CYBOCS, moderate (72%) and small (58%) effect sizes, respectively, were found. For the Anxiety Disorders Interview Schedule - Clinician Severity Rating (ADIS-CSR), the effect size was moderate, with 71% reduction (CBT + DCS) versus 53% reduction (CBT + Placebo).

Kushner et al. [29] randomized 25 participants to 10 doses of DCS (125 mg) or placebo, about 2 hours before each of 10 sessions of exposure and response prevention (twice weekly). Participants were included according to DSM-IV criteria for OCD and for having Yale-Brown Obsessive Compulsive Scale (Y-BOCS) [30] scores ≥18. The evaluations were performed at baseline, after the fourth session, at the last session and at 3-month follow-up. After the fourth session, obsession-related fears declined more rapidly in the DCS than in the placebo group, according to Y-BOCS scores. The DCS group achieved >50% reduction on the Subjective Unit of Distress Scale (SUDS) in all items of the hierarchy in two sessions earlier than the placebo group. However, after the last session of exposure and at the 3-month follow-up, there was no statistically significant difference between the two groups according to the Y-BOCS and SUDS scales, suggesting that the effect of DCS was concentrated in the first exposure sessions.

Wilhelm et al. [31] randomized 22 patients to DCS (100 mg) (n = 10) or placebo (n = 12) administered one hour before each of 10 individual sessions (60 minutes each) of behavioral therapy, twice a week. Before the protocol, participants were interviewed for a diagnosis, pre-treatment evaluation, and a session of psychoeducation/planning of the treatment. The DCS group had a significant improvement in OCD symptoms assessed by the Y-BOCS [30] as compared with the placebo group at mid-treatment (after session 5), with large effect size. However, there was no statistically significant difference between groups after treatment and at the 1-month follow-up. Also, the DCS group showed significant reduction of depression symptoms at the end of treatment, with large effect size. However, there was no statistically significant difference between the groups at mid-treatment and 1-month follow-up.

Storch et al. [32] found no statistically significant difference between the comparison groups although reduction of symptoms occurred in both groups. For this study, 24 participants were randomized to exposure and response prevention (E/RP) + DCS (250 mg) versus E/RP + placebo, administered 4 hours before each of 12 weekly sessions of E/RP (75–90 minutes each). Remission was defined as having reached gravity ≤ 3 on the Anxiety Disorders Interview Schedule for Diagnostic and Statistical Manual of Mental Disorders-IV (ADIS-IV) and ≤ 10 on the Y-BOCS [30]. There was no significant difference in remission between the groups: 42% (E/RP + DCS) and 58% (E/RP + placebo) meeting criteria for remission. The groups did not differ on the Clinical Global Improvement Scale (CGI-S) (Guy, 1970), with 83% (E/RP + DCS) and 92% (E/RP + placebo) considered as responders. Follow-up (12 months) also did not show statistically significant differences.

Three controlled trials with patients with specific phobia were identified: two with acrophobia and one with snake phobia. One of the studies with acrophobia reported positive results. The other study with acrophobia and the one study with snake phobia reported negative results.

The study by Ressler et al. [33] was the first to use DCS to facilitate extinction of fear in humans; it was also the only one that used virtual reality and psychophysiological measures. Twenty-seven participants were randomized to three groups: placebo plus Virtual Reality Exposure (VRE) therapy (n = 10), 50 mg of DCS plus VRE therapy (n = 8), or 500 mg of DCS plus VRE therapy (n = 9). The two sessions of behavioral exposure therapy were performed using virtual reality: exposure to height within a virtual glass elevator. The doses of DCS or placebo were administered acutely prior to each of the two sessions. Besides subjective and psychometric measures, an objective measure of fear electrodermal skin fluctuation was used as well. Participants who received exposure therapy plus DCS showed significantly greater reduction in symptoms of acrophobia in almost all primary outcome measures - Acrophobia Questionnaire with Avoidance (AAVQ) and Attitudes Toward Heights Inventory (ATHI) [34]. There was no statistically significant difference in the outcomes between the group that received 50 mg and the group that received 500 mg. The participants who received DCS showed higher proportions of individuals who reported “much improvement” or “very much improvement”, significant reduction in number of skin conductance fluctuations per minute of virtual exposure and significantly greater improvements in measures of symptoms of acrophobia in the real world. Improvements in subjective and objective measures were maintained at 3-month follow-up.

Tart et al. [35] replicated the procedures of the first study and randomized 29 participants with height phobia for two sessions of VRE in combination with 50 mg of DCS or placebo. This was the first study, identified in this review, using DCS immediately after the exposure session. The VRE protocol used was the same as in the previous study by Ressler et al. [33]. Participants were selected according to the DSM-IV-TR criteria for acrophobia and a subjective distress score (SUDS) >50 on the Behavioral Avoidance Test (BAT) [34]. Participants using other psychotropic medications or psychotherapy and previous non-response to exposure therapy for acrophobia were excluded. Response was defined as “very much improved” or “much improved” on CGI-I (score ≤ 2). Remission was defined as “normal” or “minimally ill” on CGI-S (score ≤ 2). Evaluations were performed at baseline, at each treatment session, one week post-treatment and at 1-month follow-up. Significant improvements were observed in all measures, with no differences between the groups. The improvement was significant in all outcome measures, but there was no significant statistical difference between the groups on DCS versus placebo on BAT, AAVQ, and CGI scores and other measures. The proportions of responders were 81.8% in the placebo group and 66.7% in the DCS group after treatment, and 80.1% in the control group and 75.0% in the DCS group at 1-month follow-up. Regarding the proportion of patients achieving remission, the authors found 63.5% in the placebo group and 60.0% in the DCS group after treatment, and 63.4% in the placebo group and 66.6% in the DCS group at 1-month follow-up.

In the study by Nave et al. [36], 20 adults with snake phobia received 50 mg of D-cycloserine or placebo 1 hour prior to a single session of graded exposure therapy. All of the participants achieved score ≥18 on the Snake Questionnaire [37], had not undergone treatment for snake phobia previously, and were suitable for functional magnetic resonance imaging (fMRI). Participants were assessed one week before and after treatment through clinical examination and the Snake Questionnaire, CGI-S, CGI-I and Snake-Stimuli Symptom Provocation Functional Magnetic Resonance Imaging Task. The DCS and placebo group responded well to the treatment, although the DCS group reached the top of the exposure hierarchy more quickly.

Two controlled studies were identified involving the combined use of CBT and DCS in the treatment of panic disorder with or without agoraphobia. One found positive results and the other found weak results for enhancement with DCS.

In Otto et al. [38], patients with or without agoraphobia and panic disorder severity of at least 4 (moderate severity) on the CGI-S were selected for 5 sessions of CBT (interoceptive exposure, cognitive, and situational exposure interventions). This was the first study using DCS for a treatment protocol emphasizing exposure to feared internal sensations (interoceptive exposure). Thirty-one participants were randomized to 50 mg of DCS or placebo, administered one hour before 3–5 sessions of CBT. Most study participants were taking psychoactive medications at stable doses for at least two months before entering the trial without positive results. Soon after the end of treatment and at 1-month follow-up, the group that received DCS instead of placebo showed better results on the Panic Disorder Severity Scale (PDSS) [39] and CGI-S, changes which were clinically significant (DCS = 77%, placebo = 33%), and larger effect size. Treatment gains were maintained at 1-month follow-up, although the difference between the DCS group and the placebo group with regard to participants meeting criteria for clinically significant change was no longer significant at follow-up (DCS = 75% and placebo = 53%). The study suggests that DCS was effective for participants who failed to respond adequately to the traditional drug treatment for panic disorder.

Siegmund et al. [40] randomized 39 participants with panic disorder and agoraphobia. All of them received 11 CBT sessions (8 sessions of 90 minutes each of CBT in a group setting+3 sessions of individual exposure in vivo). This is the only study that used flooding. One hour before the beginning of each exposure session, patients received 50 mg of DCS (n = 20) or placebo (n = 19). After randomization, the baseline assessment showed differences between the groups in three secondary measures; in all of them, the DCS group had less severity than the placebo group. However, comorbidities were significantly higher in the placebo group. Both groups improved after treatment and there was no statistical difference in the primary outcome measure – Panic and Agoraphobia Scale (PAS) [41], or on any of the secondary outcome measures. However, subsequent evaluation showed a statistical tendency to greater reduction of the PAS score in the DCS group. DCS seems to have accelerated the reduction of symptoms with exposure therapy in participants with more severe panic disorder and agoraphobia.

We identified two studies of patients with social anxiety disorder which found positive response to the enhancement with DCS.

Hofmann et. al. [42] found a positive response for the use of short-term dosing of DCS as an adjunctive intervention to exposure therapy. Twenty-seven participants with significant public speaking anxiety were randomized to DCS (50 mg) + 5 sessions of exposure therapy (n = 12) versus placebo+5 sessions of exposure therapy (n = 15). Participants received 5 weekly therapy sessions in individual or group format. DCS or placebo was administered one hour before sessions 2–5. The diagnosis of social anxiety disorder was performed using the Anxiety Disorders Interview Schedule for DSM-IV [43] and the Structured Clinical Interview for DSM- IV. The group that received DCS showed a statistically significantly greater reduction of the general symptoms of social anxiety, assessed through the questionnaires Social Phobia and Anxiety Inventory (SPAI) [44] and Liebowitz Social Anxiety Scale (LSAS) [45], with medium to large effect size (Cohen d); the improvements were maintained at one-month follow-up. They computed controlled effect sizes by dividing the differences between the mean change of the DCS group and the mean change of the placebo group by the pooled standard deviation.

Guastella et al. [46] replicated the study of Hofmann et al. [42] and developed a study of a sample that was two times larger than in previously published studies: 56 patients were randomized to 50 mg of DCS (n = 28) or placebo (n = 28). Participants were given five group sessions (90 minutes each) of an exposure protocol. In sessions 2 through 5 they received the capsules to be taken one hour before each exposure session. Participants were diagnosed with social anxiety disorder using the Anxiety Disorder Interview Schedule for Adults [47]. Improvements were evaluated by SPAI, LSAs, Brief Fear of Negative Evaluation Scale (BFNE), and Life Interference Scale (LIS) [48]. There was a reduction of symptoms in both groups. However, the DCS group had greater reductions of symptoms of social anxiety disorder on LSAs, BFNE and LIS, with moderate effect size (Cohen’s d) in most of the measures used; the improvements were maintained at 1-month follow-up. The effect sizes were computed by dividing the difference between the mean change of the DCS group and the mean change of the placebo group by the pooled standard deviation. The results suggest that punctual use of DCS with 3 or 4 sessions of exposure is effective to enhance the treatment of social anxiety disorder.

Two recent studies investigating augmentation of prolonged exposure therapy for post-traumatic stress disorder (PTSD) with DCS were identified. In one of the studies, the DCS enhanced the response of the group of participants who completed all sessions. In the other study, DCS did not enhance the response to treatment.

Kleine et al. [49], from the 75 subjects with mixed traumas selected for randomization, 45 completed the treatment protocol: exposure plus DCS (n = 24) and exposure plus placebo (n = 21). Fifty mg of DCS or placebo were administered one hour before each of 10 weekly exposure sessions. Less than half of the participants (41.8%), equally distributed between the groups, were using other psychotropic medications at a stable dose. In the initial evaluation, 70.1% (47 of the participants) had at least one additional diagnosis, mostly depression (53.7%) and anxiety disorders (41.8%). There was no significant difference between the participants dropping out of the DCS group (n = 9; 27.3%) and the placebo group (n = 13; 38.2%). Response to treatment was defined as decrease from baseline ≥10 points on the Clinician-Administered PTSD Scale (CAPS) and remission was defined as a CAPS severity score <20. Thirty-four participants (50.7%) showed post-treatment response according to the CAPS and the reduction was maintained at 3-month follow-up. There was no difference between the groups regarding the frequency of remission. Nineteen participants (42.2%) were classified as early completers, obtaining recovery before session 8. The regular completers showed more severe self-reported PTSD symptoms at session 1. DCS did not potentiate the overall treatment effects, although the regular completers (the participants who completed all sessions) who received DCS showed a greater reduction in symptoms across sessions than the regular completers on placebo. The use of DCS was associated with a greater reduction of symptoms in participants who had more severe pretreatment PTSD and needed longer treatment.

Litz et al. [50] randomized 26 veterans of the Iraq and Afghanistan wars to exposure therapy plus DCS (n = 13) or to exposure therapy plus placebo (n = 13). Six sessions (60–90 min each) of exposure therapy were conducted. DCS (50 mg) was administered 30 min prior to sessions of imaginal therapy exposure (sessions 2–5). Participants were asked to arrive at least 30 minutes prior to the start of sessions for a medical evaluation, including alcohol breath analyses and to take DCS or placebo. The effect size for primary and secondary outcomes were medium to large, with exposure therapy plus placebo faring significantly better than exposure therapy plus DCS on all outcomes. At post-treatment, 36.4% of the completers in the placebo group compared with 33.3% of those in the DCS condition no longer met criteria for PTSD on the CAPS. Responder status was defined as a reduction of 10 or more points on the CAPS. At post-treatment, 50% (n = 10) of the completers met the criteria for responder status: 70% of the placebo group and 30% of the DCS group. Follow-up evaluations were carried at 3 and 6 months, where 58% and 54% of participants met criteria for responder status, respectively. At the 6-month follow-up, the treatment effects were clinically significant, with 50% of the participants in the placebo group no longer meeting criteria for PTSD and 66% meeting criteria for respondent status.

## Analysis

### Methodological quality

In Figure 2 it can be seen that the great majority of the studies presented criteria for “low risk” in the items used for the methodological quality assessment. Most presented at least one “unclear” item, and only two had “high risk” for selected items. Figure 3 shows the proportion of studies fulfilling the quality criteria for each of the selected items.

### Combining effect sizes (meta-analysis)

Figure 4 presents the forest plot for the standardized mean differences (SMD) between intervention and control groups of the included studies. The confidence interval of most studies crosses the vertical line of null effect (mean difference equal to zero), indicating that the majority of these studies did not find a statistically significant difference between DCS and placebo.

As we found no evidence of heterogeneity between the results of the different studies (I^2^ = 10% and χ^2^ not significant: p = 0.34), their SMD were pooled to estimate the overall standardized weighted mean difference. The combined measure in figure 4 shows that the group that received D-cycloserine had an average reduction of 0.34 standard deviations at the end of follow-up as compared to the placebo group. This difference was statistically significant (p = 0.001).

Some studies reported large standard deviations, suggesting asymmetry/non-normality of distribution of their means. So, we carried out a sensitivity analysis removing these estimates to evaluate the impact on the overall standardized SMD. The result thus obtained did not differ substantially from the one observed with the 13 studies (Cohen’s d = −0.26, 95% CI: −0.50 to −0.029, p = .03).

## Discussion

### Overall efficacy

The results of this meta-analysis suggest that D-cycloserine (DCS) enhances the effects of exposure therapy in anxiety disorders. The observed effect size was, however, small to moderate (Cohen’s d = −0.34). Although we included studies with different types of anxiety disorders, their findings concerning DCS efficacy showed low heterogeneity. Evidence suggests that the use of DCS is effective at low doses, used a limited number of times and at times close to exposure therapy.

Our findings go in the same direction of two other meta-analyses [51]; [52], one systematic review [53], and two other reviews on the topic [54]; [55].

In the first published review on the topic, Hofmann et al. [54] analyzed the first two studies with D-cycloserine enhancing CBT in humans and concluded that DCS facilitates the process of extinction of conditioned fear when administered in individual doses and before exposure therapy. They recognized the limitations of their conclusion, calling attention to the fact that the existing literature is composed of relatively small sample sizes and a limited number of studies. Furthermore, they pointed out that although animal and human studies were associated with significant effect sizes, human studies had a small effect size while animal studies had a large effect size.

Norberg et al. [51], in a meta-analysis of DCS augmentation of fear extinction and exposure therapy in non-animal and animal studies, concluded that “DCS is a promising tool for translational research concerned with enhancing (or reducing) NMDA receptor function as a method for improving exposure-based therapy outcomes” (p.1123). DCS showed large treatment effect in animals (Cohen’s d = 1.19) and small to moderate effect size in humans (Cohen’s d = 0.42).

One of the factors that may explain a much larger effect size of studies with animal models compared to human studies [51] is the concomitant use of other drugs, as most studies participants were taking other psychotropic medications. Although these drugs have been stably used before the beginning of studies, and their use was controlled in statistical analyses, the mechanisms of possible interaction of DCS with other psychiatric drugs are not yet entirely known; for example, continued use of the antidepressants imipramine and citalopram appears to affect the function of the glycine/NMDA receptor [56]. The only study that established use of other medication that may interfere with DCS (e.g., anticoagulants) as an exclusion criterion was the one by Kleine et al. [49], with PTSD patients. Nevertheless, a study of social anxiety disorder [54] indicated that the use of concomitant medication does not seem to have affected the results, and there is no contraindication for the use of other psychotropic medications with DCS. Another possible explanation for the smaller effect size in humans is the fact that the sample includes human individuals with comorbid disorders, mainly other anxiety disorders and depression.

There is evidence that DCS is effective when administered at low doses (50 mg), a limited number of times, and immediately before (1 or 2 hours) or after exposure therapy [51]; [53]. Our review is consistent with these findings, since most of the studies used a dose of 50 mg and short CBT protocols. Evidence indicated that DCS does not have anxiolytic properties, not being used as a first strategy, but for the therapeutic enhancement of learning [57]. In the study by Ressler et al. [33] DCS administration did not affect baseline subjective fear levels in patients who received exposure therapy with virtual reality, i.e., administration of DCS during the therapy session did not affect the level of fear or avoidance during it. Despite the evidence with regard to time, dose and protocols, the exact dose indicated for administration of DCS as strategy of potentiation is still unknown.

On the other hand, the meta-analysis performed by Bontempo et al. [52] did not find significant interference of the time of administration and number of doses in the effects of potentiation with DCS. Also, one of the reviewed studies, conducted by Ressler et al. [33], did not find difference between doses of 50 mg and 500 mg.

Most studies in our review (n = 9/13) used 50 mg, but we also observed doses as 100 mg [31], 125 mg [29], 250 mg [32] and 500 mg [33]. Lack of standardization was also found concerning time of administration: 1, 2, 4 hours before or indefinite time [33].

DCS is a partial agonist and high doses may increase the antagonistic effects in NMDA receptors, decreasing the learning effects. A study with animal model suggests that the NMDA receptor can become desensitized after prolonged exposure to DCS [58]. At high doses and/or chronic administration, this substance seems to have a paradoxical antagonistic effect on the NMDA receptor [59], resulting in reduced effect of extinction of fear in animals. Studies with animal models also indicate the rapid development of tolerance to DCS when it is administered repeatedly and at high doses [54].

The possible efficacy of a reduced number of DCS administrations can be explained by the progressive desensitization of receptors with continued use of the substance. This finding is supported by the meta-analysis of Norberg et al. [51] on the use of DCS in animals and humans, in which the rapid development of tolerance is shown. It was also found in that meta-analysis that time of administration of DCS was a predictor of effect size, and the best effects were obtained when the substance was administered immediately before or soon after exposure. Other studies with animal models also support this finding, suggesting that the effects of augmentation with DCS occur during the period of memory consolidation that occurs after exposure rather than during exposure itself [60]; [61].

In OCD, where the lack of standardization was higher, the results for enhancement with DCS were less promising. These studies used the highest doses for a prolonged period: 100 mg before 10 sessions, 125 mg before 10 sessions, and 250 mg before 12 sessions. A difference between intervention and placebo groups was found when the drug was administered 1 to 2 hours before exposure sessions, but not when administered 4 hours before [32], which may also have contributed to the negative results, since DCS seems to be more effective when used shortly before the exposure sessions [60]. Also, these findings are in accordance with studies with animal models, reinforcing the idea that DCS is effective when administered immediately before or soon after exposure [61]. In this study by Storch et al. [32], DCS was administered at a dose of 250 mg four hours before the session and for a long period of time (12 weeks). The studies that showed positive results used brief protocols. Studies conducted by Wilhelm et al. [31] and Kushner et al. [29], who used twice-weekly sessions, found significantly higher results in the DCS group at the fifth and fourth sessions, respectively, but this effect was not observed at the end of 10 sessions.

In a additional article, Chasson et. al. [62] re-analyzed data from the study by Wilhelm et al. [31] and the outcomes indicated that the group that received DCS achieved results 2.3 times faster than the placebo group and six times faster in the first half of the sessions (1 to 5) of exposure therapy, suggesting that DCS accelerates the gains of exposure in OCD. These data indicate that the effects of DCS are concentrated in the first sessions of exposure. It is possible, therefore, that the period of memory re-consolidation can be accelerated by DCS. Moreover, the findings of these studies with OCD support the idea that the NMDA receptor can become desensitized with prolonged exposure to DCS [58], and that isolated doses of medication prevent the compensatory changes at the NMDA receptor. Even though DCS did not have more robust effects, accelerating the effects of exposure has important clinical implications. Exposure and response prevention alone require more than 16 sessions to reduce OCD severity. The addition of DCS could reduce to eight the number of sessions. Reducing the required number of sessions and patients responding more quickly to treatment could have benefits to patients and society, such as decrease in treatment refusal, dropout rates, costs (the use of DCS could save over $2100 for each patient), and reduce the anxiety provoked with exposure, facilitating the adherence of treatment [62].

Other hypotheses can also justify the negative findings in OCD, such as the heterogeneity of the disorder and the fact that it is very common for a patient with OCD to be using a Serotonin Reuptake Inhibitor (SRI). In the study by Wilhelm et al. [31], 69.5% of the participants were taking a stable dose of some psychotropic medication, in most cases an antidepressant. In Kushner et al. [29], 64.3% of the group that received DCS and 58.8% in the placebo group were using some other psychotropic medication. The use of concomitant medications, usually an antidepressant, could help to explain the negative findings, as antidepressants seem to modify (desensitize) the function of the NMDA receptor.

In both studies with social anxiety disorder [46]; [54], we found a higher standardization. Both used the same protocol of 50 mg of DCS one hour before CBT sessions 2 to 5, although Hofmann et al. al. [54] used a format of individual or group sessions. Regarding studies with panic disorder [38]; [40] both used 50 mg of DCS an hour before the sessions, but differed on the number and format of CBT sessions, which may have influenced the efficacy of DCS in the study with 8 group sessions of Siegmund et al. [40].

With regard to panic disorder, Otto et al. [38] observed statistically significant positive results in the group of DCS as compared to the control group at the end of 5 sessions. These results were not confirmed in the study by Siegmund et al. [40], who found similar results in both groups after treatment. However, this study employed 11 sessions, but it can be observed that the administration of DCS produced better results in the middle of the treatment. This again suggests that the administration of DCS in brief protocols (<5 sessions) can be effective. Another possible explanation for the weak efficacy of DCS discussed in the study refers to the good response to therapy in the placebo group. The participants in the placebo group showed a reduction of symptoms of 58% (PAS) at the last evaluation. This result was not found in the other studies with DCS and suggested a floor effect preventing additional effects with DCS. Siegmund et al. [40] suggested that this effect may be due to the higher dose of psychotherapy: 8×90 min group therapy plus three individual exposures.

PTSD is the only disorder in which DCS seems to have played only a minimal role as an enhancer of CBT. The protocol used by Kleine et al. [49] does not seem to have been effective even in mid-treatment (the study used 10 sessions). This may be due to the specificity of PTSD, which is an anxiety disorder whose time of conditioning is necessarily known. For this reason, it may involve different brain mechanisms that respond differently to the action of DCS. In this study with PTSD, DCS enhanced outcomes in the subgroup of regular completers only, who are the participants who completed all sessions.

Regarding tolerability, DCS was well tolerated with no significant adverse effects found in the reviewed studies. In Hofmann et. al. [42], DCS administration had two spontaneous notifications of acute adverse effects: nightmare the night after administration and exposure session in one participant, and euphoric mood and increased energy in one participant with chronic depression. In Storch et al. [32], only three participants of the DCS group reported adverse events that were considered moderate: Increased anxiety, drowsiness and dry mouth. For the placebo group, these included drowsiness and restlessness. Kushner et al. [29] found mild gastrointestinal distress, dizziness, fatigue, and anxiety in four participants in the DCS group and “jittery feelings”, dissociation and dry lips in three participants in the placebo group. In the study by Nave et al. [36], one participant in the DCS group reported mild nausea during the hour following administration. Kleine et al. [49] were the only ones who did not find differences between the participants in the DCS and placebo groups regarding adverse effects. At high doses (500 - 1000 mg) administered chronically, DCS may cause side effects such as headache, drowsiness, confusion, tremors, memory difficulties, paresthesias, and seizure [63]. In this sense, unlike many psychotropic medications, the side-effects of DCS at low doses are minimum [26], and therefore this drug seems to be a safe alternative for enhancing CBT outcome.

### Methodological issues

Relating to the methodological quality of the included studies in this meta-analysis, most of the articles presented a low risk of bias (Figures 1 and 2). Figure 1 shows each article separately and figure 2 shows the results of summarized articles. Most part of the studies presented a low risk of bias, with one study showing a high risk of bias for incomplete outcome data [46] and one other [40] for description of relevant comorbidities. A significant number of the studies showed an unclear risk of bias.

With reference to the quality of the included studies, all of them were randomized, double-blind, placebo-controlled trials, although we found a limited number of studies and with relatively small samples sizes. The study by Guastella et al. [46] represents an exception, with a sample twice as large (n = 56) as in previous studies. Furthermore, we did not find any studies with generalized anxiety disorder (GAD). Regarding PTSD, we identified only two studies, both published recently [49]; [50]. In addition to these, we located another study using DCS for treatment of chronic PTSD [64], but in this case the aim was to investigate the isolated benefit from prolonged use of DCS, without having the main goal of enhancing exposure therapy. In that study, treatment with DCS did not result in significant improvements as compared to the placebo group. That was the first study to investigate the efficacy of the NMDA receptor modulator in PTSD treatment.

Although all studies were double-blind, no count of pills (DCS and placebo) was reported in any study. In Kushner et al. [29], ingestion of the medication at the due time was checked by telephone.

With regard to evaluation of efficacy in the long term, almost all studies had follow-up data for varying periods, including 1 week, 1 month, two months, three months, five months and six months. The results of the action of DCS did not disappear with the discontinuation of treatment and there was no report of discontinuation of the use of DCS in studies in which it was effective. In the study of DCS in OCD by Kushner et al. [29], the improvements were maintained after the last session of exposure and at the follow-up, with no significant difference between the groups treated with DCS and placebo. In Ressler et al. [33], the improvements were maintained too and the group that received DCS reported less avoidance of heights in their daily activities, after the end of treatment and even at the three-month follow-up. Although the studies presented follow-up data, longer follow-up periods are necessary at least one year so as to confirm the maintenance of the effect of extinction augmented by DCS for a sufficiently long time.

### Limitations of the present meta-analysis and recommendation (future directions)

A limitation of this review concerns the use of just three electronic databases, even though they represent the main ones. However, specialists were consulted concerning the existence of non-published studies.

We observed that no study performed a more detailed evaluation of the effects of DCS in each exposure session. We suggest that future studies assess the degree of learning that occurs in each exposure session to better assess the possible causal connection of DCS in the process of extinction. Moreover, because the optimal time and dose for efficacy of DCS has not yet been determined, studies of response control to specific doses are necessary to determine the best dose for using DCS in extinction of fear in humans.

We recommend the development of additional double-blinded randomized trials to validate the findings in this review. With the advances in research, DCS could potentially be used in clinical settings, something that may represent advantages such as cost-efficacy, reduction of dropout and refusal rates, increased access to health care, with more patients gaining access to treatment, acceleration of treatment response with exposure therapy and development of more effective treatments for the general population. According to Otto et al. [65], 5–20% of subjects receiving CBT in randomized, controlled trials drop out of treatment. The drop-out rates for traditional drug treatments of anxiety disorders are higher. Future studies could investigate the relationship of DCS with decreases in the number of drop-out cases.

Further studies are also needed to investigate predictors of response, to determine whether the efficacy of DCS varies according to the type of anxiety disorder, dose or time of administration of DCS and CBT, to determine the long-term results of augmentation with DCS and to assess the effectiveness of the drug and exposure therapy in the real world. It would also be important to evaluate the efficacy of DCS from the psychophysiological point of view, together with the psychometric one, such as in the study of Ressler et al. [33], who evaluated skin conductance during exposure to fear of heights, concluding that the decrease in this parameter was associated with treatment efficacy. This mode of assessment of efficacy has the advantage of being more objective, not suffering interference from subjective biases, improving treatment through the discovery of mechanisms of action of the therapy and preventing the development of disorders [66]. Indeed, the development of biomarkers is crucial to the advance of behavioral treatment research [67].

We located only one study with children, which suggest that this is an area that requires additional studies. This study was the one by Storch et al. [68] with children and adolescents with OCD, and it found moderate effects and did not find significant difference between the group that received DCS and the placebo group.

It is also necessary to investigate the efficacy of DCS for individuals who did not respond sufficiently to exposure therapy. The effects of augmentation occur during the period of memory consolidation, which occurs after the exposure training. Evidence suggests that DCS would enhance the consolidation of emotional learning of exposure therapy. Thus, to maximize the effects of treatment in anxiety disorders, it is important to consider individual differences, including the level of response to exposure therapy achieved in each session so that administration of the drug occurs after successful sessions [69]; [70]. The effect of DCS seems to potentiate whatever emotional learning has occurred, so there is evidence that DCS can enhance adverse reconsolidation effects, resulting in a poorer outcome relative to placebo. There is some evidence which suggests that the post-session administration of DCS to avoids the possibility of DCS enhancing the reconsolidation of fear memory in cases of unsuccessful therapy. Moreover, the decision to administer DCS would be made post-session, dependent on the level of fear reduced by the end of the exposure session [71]. This evidence could clarify the decision on the use of DCS and the best dose timing. The investigation of the use of DCS after successful and unsuccessful sessions is necessary to support these findings.

## Supporting Information

Checklist S1 **PRISMA checklist**.
(DOC)
